# H3K79me2/3 controls enhancer–promoter interactions and activation of the pan-cancer stem cell marker *PROM1*/CD133 in MLL-AF4 leukemia cells

**DOI:** 10.1038/s41375-020-0808-y

**Published:** 2020-04-02

**Authors:** Laura Godfrey, Nicholas T. Crump, Sorcha O’Byrne, I-Jun Lau, Siobhan Rice, Joe R. Harman, Thomas Jackson, Natalina Elliott, Gemma Buck, Christopher Connor, Ross Thorne, David J. H. F. Knapp, Olaf Heidenreich, Paresh Vyas, Pablo Menendez, Sarah Inglott, Philip Ancliff, Huimin Geng, Irene Roberts, Anindita Roy, Thomas A. Milne

**Affiliations:** 1 MRC Molecular Haematology Unit, MRC Weatherall Institute of Molecular Medicine, NIHR Oxford Biomedical Research Centre Haematology Theme, Radcliffe Department of Medicine, University of Oxford, Oxford, UK; 2 Department of Paediatrics, University of Oxford, Oxford, UK; 3 Great Ormond Street Hospital for Children, London, UK; 4 MRC Weatherall Institute of Molecular Medicine, Radcliffe Department of Medicine, University of Oxford, Oxford, UK; 5 Princess Maxima Centrum for Pediatric Oncology, Utrecht, The Netherlands; 6 Wolfson Childhood Cancer Research Centre, Newcastle University, Newcastle upon Tyne, UK; 7 Department of Haematology, Oxford University Hospitals NHS Foundation Trust, Oxford, UK; 8 Josep Carreras Leukemia Research Institute, Barcelona, Spain; 9 Institucio Catalana of Recerca i Estudis Avançats (ICREA), Barcelona, Spain; 10 Centro de Investigación Biomédica en Red en cancer (CIBERONC)-ISCIII, Barcelona, Spain; 11 Department of Laboratory Medicine, University of California, San Francisco, San Francisco, CA 94143 USA

**Keywords:** Acute lymphocytic leukaemia, Cancer genomics

## Abstract

MLL gene rearrangements (MLLr) are a common cause of aggressive, incurable acute lymphoblastic leukemias (ALL) in infants and children, most of which originate in utero. The most common MLLr produces an MLL-AF4 fusion protein. MLL-AF4 promotes leukemogenesis by activating key target genes, mainly through recruitment of DOT1L and increased histone H3 lysine-79 methylation (H3K79me2/3). One key MLL-AF4 target gene is *PROM1*, which encodes CD133 (Prominin-1). CD133 is a pentaspan transmembrane glycoprotein that represents a potential pan-cancer target as it is found on multiple cancer stem cells. Here we demonstrate that aberrant *PROM1*/CD133 expression is essential for leukemic cell growth, mediated by direct binding of MLL-AF4. Activation is controlled by an intragenic H3K79me2/3 enhancer element (KEE) leading to increased enhancer–promoter interactions between *PROM1* and the nearby gene *TAPT1*. This dual locus regulation is reflected in a strong correlation of expression in leukemia. We find that in *PROM1*/CD133 non-expressing cells, the *PROM1* locus is repressed by polycomb repressive complex 2 (PRC2) binding, associated with reduced expression of *TAPT1*, partially due to loss of interactions with the *PROM1* locus. Together, these results provide the first detailed analysis of *PROM1*/CD133 regulation that explains CD133 expression in MLLr ALL.

## Introduction

CD133, encoded by the *PROM1* gene, is a pentaspan transmembrane glycoprotein of great potential value as a pan-cancer target as it is commonly associated with cancer stem cells in multiple different tumor types, including leukemia [1, 2]. Proof-of-principle studies have shown that targeting CD133 can be used to deliver nanoparticles to gastric stem cells [3], or for chimeric antigen receptor T cell therapy in acute lymphoblastic leukemias (ALL) caused by rearrangements of the *Mixed Lineage Leukemia (MLL)* gene [4].

Despite vast improvements in treatment for ALL, *MLL* gene rearrangements (MLLr) still cause very poor prognosis ALLs especially in infants [5–9]. The most common MLL rearrangement is the t(4;11) (q21;q23) chromosome translocation that fuses MLL in frame with the AF4 gene producing MLL-AF4 and AF4-MLL fusion proteins [7, 10–12]. MLL-AF4 and other MLL fusion proteins (MLL-FPs) bind to gene targets and cause inappropriate gene activation through multiple transcription elongation and epigenetic mechanisms [13], including recruitment of the histone H3 lysine-79 (H3K79) methyltransferase DOT1L [7, 14–17]. In addition to a role in transcription elongation, recent work from our lab has shown that H3K79me2/3 has an important role at a subset of enhancers (H3K79me2/3-marked enhancer elements (KEEs)), increasing expression of key gene targets through the maintenance of enhancer–promoter interactions [18].

One of the most attractive features of *PROM1*/CD133 as a potential therapeutic target derives from the recognition that the gene is a direct target of MLL-AF4 regulation [17, 19, 20], suggesting that in MLLr leukemias *PROM1*/CD133 expression is tightly linked to the activity of the fusion protein itself. However, the exact details of how this locus is regulated by MLL-AF4 is unclear, and whether and how *PROM1*/CD133 contributes to MLLr leukemic growth is unknown. Understanding these mechanisms is likely to be key to the future development of *PROM1*/CD133-directed therapeutic targeting in these leukemias.

To better understand the role of *PROM1*/CD133 in MLLr leukemias, we have systematically characterized the structure of the *PROM1* gene locus and the mechanism of *PROM1*/CD133 regulation for the first time. Importantly, we show that two CD133-expressing MLL-AF4 cell lines (SEM and RS4;11) are addicted to its continued expression. Using a high-resolution chromosome conformation capture technique (next generation Capture-C [21]) to analyze the enhancer–promoter structure of the *PROM1*/CD133 locus, we find that *PROM1* expression is activated by a series of intragenic KEEs within *PROM1* as well as in the nearby *TAPT1* gene. As with other well-characterized KEEs [18], we demonstrate that MLL-AF4 aberrantly upregulates *PROM1* transcription by controlling enhancer–promoter interactions via increased H3K79 methylation and H3K27 acetylation. In contrast, we find that in CD133-negative leukemia cells the *PROM1* promoter is bound by components of the polycomb repressive complex 2 (PRC2) and this is associated with a lack of enhancer features, including enhancer–promoter interactions. Overall, our results show that *PROM1*/CD133 expression is directly upregulated by the presence of MLL-AF4 through H3K79me2/3-mediated enhancer–promoter interactions, and that *PROM1* expression contributes to the leukemic growth of CD133+ cells. These results for the first time elucidate the mechanism of *PROM1*/CD133 upregulation and provide an explanation for the widespread expression of CD133 in MLLr ALLs. It also provides an interesting paradigm illustrating how repression of a single locus (*PROM1*) can impact regulation of a nearby locus (*TAPT1*) by suppressing access to key enhancer sequences.

## Materials and methods

### Cell culture and cell lines

SEM (MLL-AF4 B-ALL line [22]), ML-2 (MLL-AF6 AML line [23]), SHI-1 (MLL-AF6 AML line [24]), and RCH-ACV (E2A-PBX1 cALL line [25]) cells were purchased from DMSZ (www.cell-lines.de) and cultured in Iscove’s Modified Dulbecco’s Medium (IMDM) supplemented with 10% fetal calf serum (FCS, Gibco) and Glutamax (ThermoFisher Scientific). RS4;11 (MLL-AF4 B-ALL line), THP1 (MLL-AF9 AML line), MV4;11 (MLL-AF4 AML line), and CCRF-CEM (T-ALL line) cells were purchased from ATCC (www.lgcstandards-atcc.org). RS4;11, THP1, and CCRF-CEM were cultured using RPMI with 10% FCS and Glutamax. MV4;11 cells were cultured as per the SEM conditions. All cell lines were confirmed free from mycoplasma contamination.

### Cell culture drug treatment

Cells were treated with EPZ-5676 as previously described [17, 18]. Briefly, SEM and RS4;11 cells were treated with 2 µM EPZ-5676 or dimethyl sulphoxide (control). Cells were treated for 7 days with media and EPZ-5676 changed on days 3 and 6 and diluted to 0.5 × 10^6^ cells/ml and 0.8 × 10^6^ cells/ml, respectively. Cells were collected on day 7 and processed for downstream applications. Cells were treated for 5 days with UNC1999 at 5 µM and GSK126 at 2 µM, with cells split and fresh media and inhibitor added on day 3. Venetoclax was used at 0.5 µM for 24 h, and camptothecin was used at 1 µM for 1 h.

### Primograft

For details of sample preparation see Ref. [17].

### Patient samples

Infant and pediatric ALL samples were obtained from patients being treated at Great Ormond Street Hospital for Children, London after informed consent, and analyzed as part of their diagnostic workup. Additional MLLr ALL samples were obtained from Bloodwise Childhood Leukaemia Cell Bank, UK (REC: 16/SW/0219). All ALL samples were anonymised at source, assigned a unique study number and linked. Donated fetal tissue was provided by the Human Developmental Biology Resource (www.hdbr.org, covered under REC: 08/H0906/21+5) regulated by the UK Human Tissue Authority (www.hta.gov.uk). Second-trimester fetal bone marrow (BM) and liver was processed as previously described [26]. CD34+ and CD34− populations were isolated using magnetic beads (Miltenyi).

### Flow cytometry analysis

Cells were stained with fluorophore-conjugated monoclonal antibodies in PBS with 2% FBS and 1 mM EDTA for 30 min and analyzed using BD LSR II or Fortessa X50 instruments. For viability eBioscience Fixable Viability Dye eFluor 506 (ThermoFisher Scientific) or Hoechst 33342 (ThermoFisher Scientific) was used. Annexin V binding was assayed using the eBioscience Annexin V Apoptosis Detection Kit APC (ThermoFisher Scientific). Antibodies used are detailed in Supplementary Table 1. Analysis was performed using FlowJo software with gates set using unstained and fluorescence-minus-one controls.

### siRNA knockdown

siRNA knockdowns were performed in SEM and RS4;11 cells as described [17]. For *PROM1* KD, Ambion Silencer select siRNAs (ThermoFisher Scientific) were used: **siRNA 1**: s16879; **siRNA 2**: s16879; **NT**: Silencer Negative Control No. 2. For MLL-AF4 KD, **NT siRNA**: sense AAAAGCUGACCUUCUCCAAUG; antisense CAUUGGAGAAGGUCAGCUUUUCU. **MLL-AF4 KD siRNA**: sense AAGAAAAGCAGACCUACUCCA; antisense UGGAGUAGGUCUGCUUUUCUUUU.

### SEM *PROM1*/CD133 shRNA lines

shRNA sequences, which were designed using the siRNA targeting sequences, were cloned into a doxycycline-inducible shRNA vector (miRE_18_LT3GEPIR) using the following sequences. ***PROM1*** **shRNA 1:** GAAGGTATATTGCTGTTGACAGTGAGCGGTTTACCGCAAAAATTAGAGCCTAGTGAAGCCACAGATGTAGGCTCTAATTTTTGCGGTAAAATGCCTACTGCCTCGGACTTCAAGGGGCTAG; ***PROM1*** **shRNA 2:** TCGAGAAGGTATATTGCTGTTGACAGTGAGCGGCATTTGTTACAGCAACGACACTAGTGAAGCCACAGATGTAGTGTCGTTGCTGTAACAAATGTTGCCTACTGCCTCGGACTTCAAGGGGCTAG. SEM cells were lentivirally transduced with either shRNA plasmid and clonal lines were established using 0.5 µg/ml puromycin.

### Colony-forming assay

Wild-type or *PROM1* shRNA SEM cell lines were treated with 0.5 µg/ml doxycycline 24 h prior to colony assay plating. Five hundred doxycycline-treated or -untreated cells were plated in IMDM MethoCult media with 20% FCS (H4100; STEMCELL Technologies) per dish, in triplicate, with 0.5 µg/ml doxycycline added to the media where appropriate. Colonies were incubated for 14–16 days (37 °C, 5% CO_2_) and then counted. For replating, after counting cells were washed in IMDM, and 500 cells were added to fresh IMDM MethoCult media, with doxycycline added where appropriate, and plated for 14 days.

### Cell cycle analysis

1 × 10^6^ cells were fixed in 70% ethanol and incubated at 4 °C overnight, then stained with FxCycle PI/RNase staining solution (ThermoFisher Scientific). Cells were analyzed using 532 nm excitation and collected using 585/42 bandpass. Analysis was performed using FlowJo software.

### Immunofluorescence

Cells were prepared for imaging by attachment to coverslips coated with 0.01% Poly-L-Lysine and fixed for 15 min with 4% PFA. Cells were incubated with anti-γH2AX primary antibody (Cell Signaling Technology 9178) for 1 h at room temperature. Cells were then washed with PBS and incubated with secondary antibody for 30 min at room temperature. Cells were washed with PBS and mounted onto slides using Vectashield with DAPI. Images were acquired at room temperature using a Zeiss AXIO Observer.Z1 inverted microscope equipped with a Zeiss LSM‐880 confocal system using a Plan-Apochromat 63x/1.4 Oil DIC M27 objective. Images were then processed using OMERO (v5.4.9).

### Western blotting

Salt-soluble protein and histone extraction from 1 × 10^6^ SEM cells was conducted as previously described [18], followed by western blotting [27]. Antibodies used are detailed in Supplementary Table 1.

### qRT-PCR

For qPCR applications, RNA was extracted from 1 × 10^6^ cells using the RNeasy Mini Kit (Qiagen). RNA was reverse transcribed using Superscript III (ThermoFisher Scientific) with random hexamer primers (ThermoFisher Scientific). Samples were analyzed by Taqman qPCR, using the housekeeping gene *YWHAZ* for gene expression normalization. For list of qPCR primers used see Supplementary Table 2.

### Poly(A) RNA purification and sequencing

RNA was extracted from 1 × 10^7^ *PROM1* shRNA 1 SEM cells lines using the Directzol RNA MiniPrep Kit (Zymo R2050). Poly(A) purification was conducted using the NEB Poly(A) mRNA magnetic isolation module following the manufacturer’s protocol. Library preparation was carried out using the Ultra II Directional RNA Library Prep Kit (NEB, E7765). RNA libraries were sequenced by paired-end sequencing using a 150 cycle high output kit on a Nextseq 500 (Illumina).

### Gene expression analysis

Following sequencing, QC analysis was conducted using the fastQC package (http://www.bioinformatics.babraham.ac.uk/projects/fastqc). Reads were mapped to the human genome assembly hg19 using STAR [28]. PCR duplicates were removed from the mapped reads using Samtools [29]. The featureCounts function from the Subread package was used to quantify gene expression levels using standard parameters. This was used to identify differential gene expression globally, generating RPKM values, using the edgeR package [30]. Differential gene expression was defined by an adjusted *p* value (FDR) of <0.05. Infant ALL [31–33] and normal BM [34–36] RNA-seq datasets were analyzed as described previously [36].

### Single-cell gene expression analysis

Single-cell data was analyzed using the Seurat package [37] following standard methods. Briefly, a random subset of 10^5^ cells were chosen for analysis. Cells were initially filtered removing those that contained less than 200 and more than 2500 genes. Cells with >5% mitochondrial genes were removed. Genes detected in less than four cells were removed. Data were normalized using the log normalize method with a scale factor of 10,000. FindVariableFeatures, RunPCA, and RunTSNE were used for dimension reduction. FindNeighbours and FindCluster functions were used to find cell clusters. FindAllMarkers was used to marker genes of each cluster and cell types were identified using published marker signatures [38] and a custom gene voting system. Cell clusters and gene expression were visualized using DimPlot and FeatureScatter functions.

### ChIP-qPCR and ChIP-seq

The full protocol is described in [17, 18]. Briefly, 10^7^–10^8^ cells were sonicated (Covaris) following the manufacturer’s protocol. Magnetic protein A and G beads (ThermoFisher Scientific) were used to isolate antibody-chromatin complexes. Antibodies used are detailed in Supplementary Table 1. Beads were washed three times using a solution of 50 mM HEPES-KOH (pH 7.6), 500 mM LiCl, 1 mM EDTA, 1% NP40 and 0.7% sodium deoxycholate, and once with Tris-EDTA. Samples were eluted and Proteinase K/RNase A-treated. Samples were purified using the Qiagen PCR purification kit. DNA content was analyzed by qPCR or ChIP-sequencing. For list of qPCR primers used see Supplementary Table 2. For ChIP-seq, DNA libraries were generated using the NEBnext Ultra DNA library preparation kit for Illumina (NEB). Libraries were sequenced by paired-end sequencing using a 75 cycle high output kit on a Nextseq 500 (Illumina).

### ATAC-seq

ATAC-seq was performed using the Nextera Tn5 transposase (Illumina) on 6 × 10^4^ THP1 cells and whole BM from an MLL-AF4 ALL pediatric patient, as previously described [18, 39]. Libraries were sequenced by paired-end sequencing using a 75 cycle high output kit on a Nextseq 500 (Illumina).

### Sequence analysis

For ChIP-seq and ATAC-seq, quality control of FASTQ reads, alignment, PCR duplicate filtering, blacklisted region filtering, and UCSC data hub generation was performed using an in-house pipeline (https://github.com/Hughes-Genome-Group/NGseqBasic/releases) as described [18]. The Homer tool makeBigWig.pl command was used to generate bigwig files for visualization in UCSC, normalizing tag counts to tags per ten million. Gene profiles were generated using annotatePeaks.pl.

### Capture-C

For details on Capture-C methods used see Refs. [18, 21]. Biotinylated oligo probes, with the following sequences, were designed to the *PROM1* and *TAPT1* promoters. **PROM1_F:** GCGGCTTCCTCTGTCCCGGACGGGGACCTAGGTATGGGGCCGCCGTAATGGAAAGGATTCCTTAAACATACTCACCGCGGCGGGAGAGCTGAGAGCATGGCCAGGTGCCGCGTGGGGATC; **PROM1_R**: GATCCCGAGCCTCTGACTTTCTACAGCCGTGAAGCTCCTGGTCTGTCCACGCTCCTCTTTGTTGTCCGGTCGGCGTGTTCTGGCAGGGTGCGCCTTGAGCACCCCAGTTCTCCATCTAGC; **TAPT1_F**: GATCAACTTTATGCCCCTTAAGTAGTTTTAAAATTTCAGTACTCAAGTACAATCAATGATGTAATCAGCCTCACAACTTAGTTATTTGCATGAACTTCAGGAAATAACGGCTAACACAGC;**TAPT1_R:** GTGTCCGGCGGTCCGCTCAGGGCCTCTTTTGCAGACTCGGTGCCCGGAGTGCGCCGGCGCCGCCCGCCAGGTCTTGGCACTGCTGGCGCGGCCGCGGCGGCGGGGGCCCGGCTCCAGATC. Data analysis was performed using an in-house pipeline (https://github.com/Hughes-Genome-Group/CCseqBasicF/releases; Davies 2016) and statistical analysis was performed as described [18].

### Statistical analysis

Statistical analyses used and sample sizes are indicated in figure legends; *n* numbers refer to independent experiments (for cell lines) or biological samples (for patient data). All tests were conducted two-tailed. Samples were only excluded from analysis if positive and negative controls did not give the expected results.

## Results

### MLLr leukemia cell lines and primary ALL blasts show heterogeneous expression of *PROM1*/CD133

**Fig. 1:**
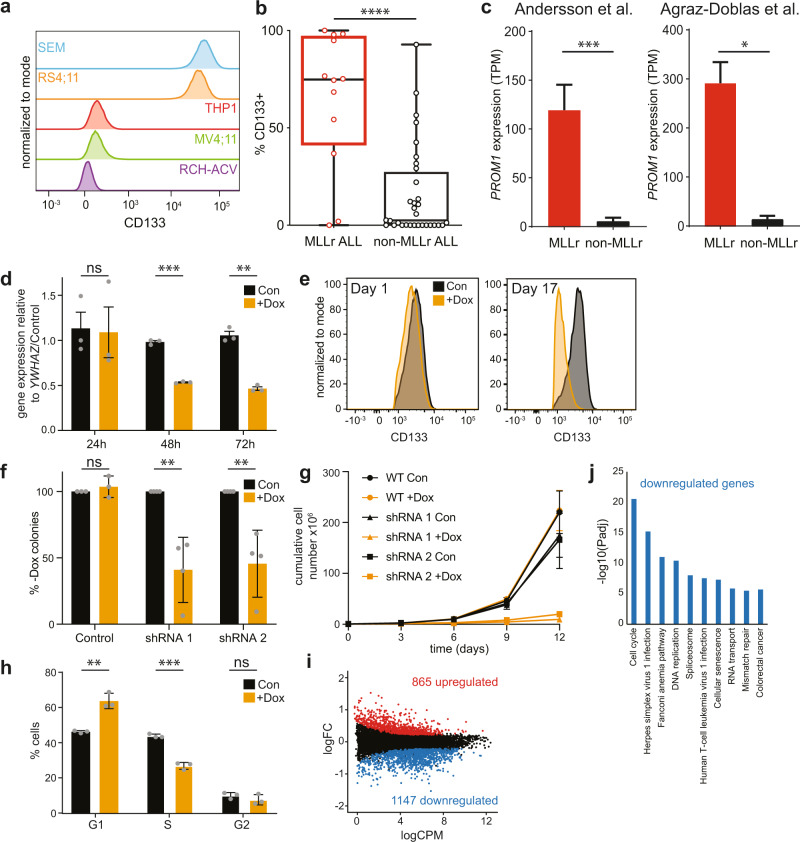
CD133 expression is essential for MLL-AF4 ALL cell growth. **a** Flow cytometry data showing CD133+ expression in different leukemia cell lines. Representative of two biological replicates. **b** Flow cytometry data showing the percentage of CD133+ cells in blast populations from 12 MLLr and 32 non-MLLr infant and childhood ALL patients. Box represents median and interquartile range; whiskers show minimum and maximum values, ****p < 0.0001. Mean values: 64.9% ± 10.2 s.e.m. and 15.7% ± 4.2 s.e.m.; median: 74.9% and 2.5% for MLLr and non-MLLr blasts, respectively. **c** Expression of *PROM1* by RNA-seq from two independent published datasets Left: expression of *PROM1* in MLL-AF4 ALL patients compared with non-MLLr ALL patient blasts from Ref. [32], ***p < 0.001; right: expression of *PROM1* in MLLr ALL patients compared with non-MLLr ALL patient blasts from Ref. [33], *p < 0.05. **d** qRT-PCR of *PROM1* in untreated control (black) and doxycycline-treated (orange) *PROM1* shRNA 1 SEM cells after 24, 48, and 72 h. Error bars represent s.e.m. of three biological replicates, ***p < 0.001, **p < 0.01, ns = no significant difference. **e** Flow cytometry analysis showing CD133 level in *PROM1* shRNA 1 SEM cell line in control (black) and induced (orange) conditions at day 1 (24 h doxycycline treatment) and day 17 (following colony assay). Histograms are representative of three replicates. **f** Colony assay showing percentage of colonies formed in control and *PROM1* shRNA 1 and 2 SEM cell lines in the presence of 0.5 µg/ml doxycycline (+Dox) compared with uninduced control (Con = −Dox). Error bars represent s.d. of three (control) or four (shRNA) biological replicates, **p < 0.01, ns = no significant difference. **g** Growth curve showing cumulative cell number for control (circles) and *PROM1* shRNA 1 (triangles) and 2 (squares) SEM cell lines grown in the presence (+Dox, orange) or absence (Con, black) of 0.5 µg/ml doxycycline. Cells were split into fresh medium every 3 days. Error bars represent s.e.m. of three biological replicates. **h** Cell cycle analysis using flow cytometry in control (black) and 72 h post induction (+Dox, orange) of *PROM1* shRNA 1 cell line. Error bars represent s.e.m. from three biological replicates, ***p < 0.001, **p < 0.01, ns = no significant difference. **i** MA plot showing differentially expressed genes from RNA-seq 72 h post induction of *PROM1* shRNA 1. Statistically significant differences (865 upregulated: red; 1147 downregulated: blue; and 10,385 unchanged: black) from three biological replicates, FDR < 0.05. **j** KEGG analysis of significantly downregulated genes from RNA-seq analysis, displaying ten most enriched processes.

In order to model *PROM1*/CD133 behavior and regulation, we first evaluated CD133 expression by flow cytometry to identify CD133-positive and -negative leukemia cell lines (Fig. 1a). As also observed by others [4], while two MLL-AF4 ALL cell lines (SEM and RS4;11) displayed high levels of CD133 expression, CD133 was undetectable on the surface of THP1 (MLL-AF9 AML), MV4;11 (MLL-AF4 AML), and RCH-ACV (non-MLLr ALL) cells (Fig. 1a). This was confirmed at the RNA level by qRT-PCR (Supplementary Fig. 1a). Interestingly, *PROM1* demonstrated a similar expression pattern to the neighboring gene *TAPT1* (Supplementary Fig. 1a, b). Although *TAPT1* is expressed even when *PROM1* is not (albeit at lower levels), higher *PROM1* expression correlated with higher *TAPT1* expression in the cell lines analyzed (Supplementary Fig. 1a). We observed a similar correlation of expression in a published dataset [32] of leukemia patient samples (Supplementary Fig. 1c, R^2^ = 0.4251), confirming this phenomenon is not restricted to cell lines and suggesting the genes may be co-regulated in leukemia. Cell surface expression of CD133 does not cause upregulation of *TAPT1*, as transfection of CD133-negative THP1 cells with a *PROM1* expression plasmid did not result in an increase in *TAPT1* expression (Supplementary Fig. 1d). This suggests that it is the active transcription of the *PROM1* locus itself that may contribute to the upregulation of *TAPT1*.

To determine if heterogeneity in CD133 expression was observed in ALL patient samples, we analyzed 44 primary precursor B-ALL samples: 12 MLLr and 32 non-MLLr (sorting strategy shown in Supplementary Fig. 1e). Although there was a wide range of CD133 expression (measured by the proportion of blasts with detectable CD133) in both MLLr and non-MLLr ALL, mean and median levels of CD133 expression were markedly higher in MLLr cases (Fig. 1b, *p* < 0.0001, mean 64.9% ± 10.2 s.e.m. vs. 15.7% ± 4.2 s.e.m., median 74.9% vs. 2.5% for MLLr and non-MLLr blasts, respectively; Supplementary Fig. 1f), and a significantly greater number of MLLr ALL samples (9/12) expressed CD133 on the majority of cells (>50%) compared with 4/32 non-MLLr ALL. To correlate this with gene expression patterns, we analyzed RNA-seq data from published datasets of large cohorts of infant and childhood ALL patient blasts [32, 33]. In keeping with previous findings [40], MLLr leukemia cells were found to express *PROM1* at much higher levels than the non-MLLr cohort (Fig. 1c).

To test whether the specific MLL fusion partner contributes to *PROM1* expression status we analyzed two ALL datasets for which fusion partner status was available [31, 32]. Strikingly, whilst MLL-AF4 and MLL-ENL ALL samples displayed high levels of *PROM1* expression, other fusion proteins, including MLL-AF9, were associated with lower levels of expression, more comparable to non-MLLr leukemias (Supplementary Fig. 1g). Taken together, these data indicate that although a CD133+ immunophenotype is not restricted to MLLr ALL, *PROM1*/CD133 is aberrantly expressed in a large subset of cell lines and MLLr leukemia patients, particularly those associated with MLL-AF4 and MLL-ENL fusion proteins.

### CD133 is important for MLL-AF4 ALL leukemogenesis

To test whether CD133 is important for MLL-AF4 leukemia growth we generated two SEM cell lines containing different doxycycline-inducible shRNA sequences targeting *PROM1*. Levels of *PROM1* RNA were significantly reduced 48 h after induction (Fig. 1d). To test whether *PROM1* is important for leukemogenesis we performed colony-forming assays on cells after 24 h doxycycline treatment. A reduction in CD133 expression prior to colony assay (day 1) was verified by flow cytometry (Fig. 1e and Supplementary Fig. 1h), and levels were further decreased in the cells post-colony assay (day 17). We observed a striking reduction in colony-forming ability following *PROM1* knockdown using either shRNA, and not with doxycycline-treated control SEM cells, with the number of colonies reduced to <50% of the uninduced control (Fig. 1f). This effect was reproducible following two rounds of serial replating of colonies (Supplementary Fig. 1i). Thus, CD133 is clearly an important protein for the leukemic potential of SEM cells.

The inability of *PROM1* KD SEM cells to form colonies was matched by a dramatically reduced growth rate in liquid culture (Fig. 1g). We confirmed this in another MLL-AF4 CD133+ cell line, RS4;11 (Fig. 1a), where siRNA-mediated knockdown of *PROM1* (Supplementary Fig. 2a) had a similarly dramatic effect on cell growth (Supplementary Fig. 2b). The loss of cell growth following *PROM1* KD may be explained by a reduced growth rate and/or increased apoptosis. We tested the former possibility by performing cell cycle analysis of SEM cells after shRNA induction (Fig. 1h and Supplementary Fig. 2c). This revealed a shift in cell cycle status following *PROM1* depletion, with an increase in the proportion of cells in G1 phase and a decrease in S phase, suggesting a delay in transition between the stages (Fig. 1h and Supplementary Fig. 2c). We tested for apoptosis by Annexin V staining (Supplementary Fig. 2d) and western blot for cleaved PARP (Supplementary Fig. 2e), a known caspase 3 substrate. These demonstrated an increase in apoptosis following *PROM1* shRNA induction, but not doxycycline treatment of control SEM cells (Supplementary Fig. 2d, e). Taken together, these data suggest that *PROM1* is likely involved in promoting both cell growth and survival.

To further investigate the dependence of SEM cells on CD133 expression, we performed RNA sequencing in the *PROM1* shRNA cell line. Differential gene expression analysis revealed 865 up- and 1147 down-regulated genes 72 h after *PROM1* shRNA induction (Fig. 1i). Consistent with the disrupted cell cycle of these cells, the downregulated genes were enriched for genes involved in the cell cycle and other processes associated with deregulation of the cell cycle, including DNA replication (Fig. 1j). We also observed downregulation of DNA repair genes, including genes involved in the Fanconi anemia pathway and mismatch repair (Fig. 1j), which may be a consequence of the reduced S phase (Fig. 1h) as these processes are associated with DNA replication. This was not associated with an increase in double-strand breaks, as measured by γH2AX levels (Supplementary Fig. 2f, g).

### MLL-AF4 directly binds to and regulates *PROM1* expression

**Fig. 2:**
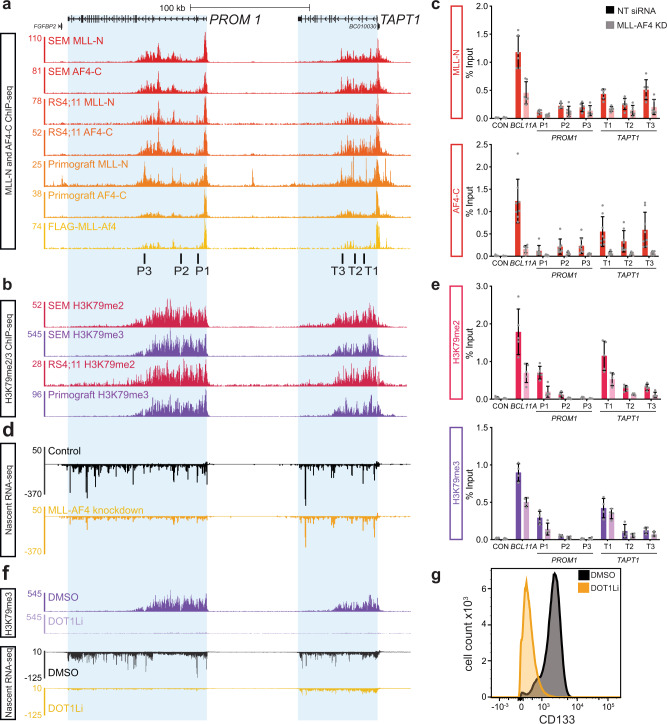
MLL-AF4 regulates *PROM1* via H3K79me2/3. **a** MLL-N and AF4-C ChIP-seq in SEM, RS4;11, MLL-AF4 ALL primograft, and FLAG-tagged MLL-Af4 cell line at *PROM1* and *TAPT1*. Location of primers used for ChIP-qPCR is indicated below the traces. **b** H3K79me2/3 ChIP-seq at *PROM1* and *TAPT1* in SEM, RS4;11, and primograft cells. **c** MLL-N and AF4-C ChIP-qPCR in control (red) and MLL-AF4 siRNA knockdown (pink) SEM cells at a negative control locus (CON), *BCL11A* and several regions of *PROM1* and *TAPT1* in SEM cells (primer locations indicated in **a**). Error bars represent s.e.m. from five biological replicates. **d** Nascent RNA-seq from control (black) and MLL-AF4 siRNA knockdown (orange) SEM cells at *PROM1* and *TAPT1*. **e** H3K79me2 and H3K79me3 ChIP-qPCR in control (dark shade) and MLL-AF4 siRNA knockdown (light shade) SEM cells at a negative control locus (CON), *BCL11A* and several regions of *PROM1* and *TAPT1* in SEM cells (primer locations indicated in **a**). Error bars represent s.e.m. from five biological replicates. **f** Reference-normalized H3K79me3 [18, 58] ChIP-seq and nascent RNA-seq in control (DMSO; dark purple/black) and EPZ-5676-treated (DOT1Li; light purple/orange) SEM cells at *PROM1* and *TAPT1*. **g** Flow cytometry analysis of CD133 level in SEM cells following control (DMSO; black) or EPZ-5676 treatment (DOT1Li; orange). Representative of two biological replicates.

To understand how *PROM1* expression is regulated in CD133+ MLLr leukemias, we analyzed ChIP-seq datasets from multiple cell types, using MLL-N and AF4-C binding as a proxy for MLL-AF4 fusion protein binding. In line with previous findings [17, 20, 27, 41], we identified MLL-AF4 binding at the promoter and spreading into the gene body of *PROM1* in CD133+ ALL cell lines (SEM and RS4;11; see Fig. 1a) and in MLL-AF4 blasts from a CD133+ ALL patient primograft [17] (Fig. 2a and Supplementary Fig. 3a). Published FLAG-MLL-Af4 ChIP-seq from MLL-Af4-transformed CD34+ cord blood cells [20] revealed a similar binding distribution at the *PROM1* locus (Fig. 2a, bottom panel). Notably, in all four cell types the neighboring *TAPT1* gene was also marked by a broad domain of MLL-AF4 binding (Fig. 2a). H3K79me2/3, a histone modification found at high levels at MLL-FP gene targets [16, 17], was observed to colocalize with MLL-AF4 at *PROM1* in SEM, RS4;11, and the MLL-AF4 ALL primograft cells, further validating the gene as a bona fide MLL-AF4 target (Fig. 2b).

Binding of MLL-AF4 to *PROM1* suggests that it may directly regulate *PROM1* transcription. To test this, we interrogated nascent RNA-seq data from SEM cells following siRNA-mediated knockdown of MLL-AF4 [17, 18], which produced clear reductions in MLL-N and AF4-C binding across *PROM1* and *TAPT1*, as well as a known MLL-AF4 gene target, *BCL11A* (Fig. 2c) [17, 18]. We observed a dramatic reduction in *PROM1* transcription following MLL-AF4 knockdown (*p* < 0.001, Fig. 2d and Supplementary Fig. 3b), confirmed by qRT-PCR (Supplementary Fig. 3c), demonstrating that MLL-AF4 is required for expression of *PROM1*. MLL-AF4 knockdown also reduced the expression of *TAPT1*, consistent with the idea that the two genes are co-regulated by MLL-AF4 (*p* < 0.001, Fig. 2d and Supplementary Fig. 3b, c). Taken together, these data demonstrate that *PROM1* is directly bound and transcriptionally regulated by MLL-AF4 in CD133+ MLL-AF4 ALL.

### MLL-AF4 controls *PROM1* expression via H3K79me2/3

We and others have shown that a common mechanism of MLL-AF4 gene activation is via aberrant recruitment of DOT1L leading to high levels of H3K79me2/3 [16, 17]. To test whether MLL-AF4 directs DOT1L activity at *PROM1* we performed DOT1L and H3K79me2/3 ChIP-qPCR in SEM cells following MLL-AF4 knockdown. We observed a reduction in both DOT1L and H3K79me2/3 at *PROM1* and *TAPT1*, as well as *BCL11A* (Fig. 2e and Supplementary Fig. 3d). This indicates that MLL-AF4 may control *PROM1* and *TAPT1* transcription via H3K79me2/3.

To test this we used the DOT1L-specific small molecule inhibitor EPZ-5676 (DOT1Li) to deplete levels of H3K79 methylation, with a near-complete loss of H3K79me3 at *PROM1* and *TAPT1* (Fig. 2f). Expression of both *PROM1* and *TAPT1* was sensitive to DOT1Li, with nascent RNA-seq [17, 18] and qRT-PCR demonstrating a significant decrease in transcription, comparable to the downregulation observed following MLL-AF4 knockdown (Fig. 2f and Supplementary Fig. 3b, c). Levels of cell surface CD133 were similarly reduced following DOT1Li (Fig. 2g). Taken together these data suggest that MLL-AF4 controls *PROM1* expression via deposition of H3K79me2/3.

### *PROM1* is regulated by H3K79me2/3 enhancers

**Fig. 3:**
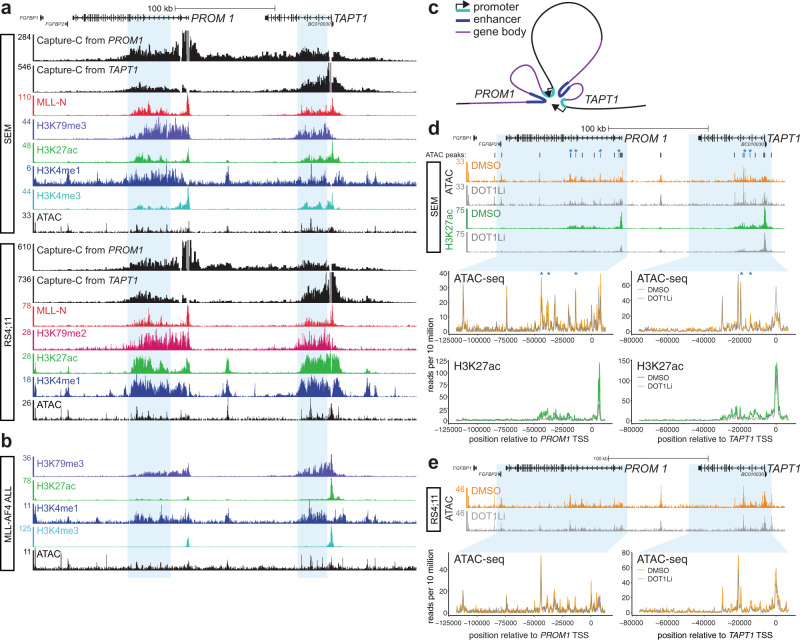
*PROM1* is regulated by H3K79me2/3-marked enhancers. **a** Capture-C tracks from the viewpoint (gray bars) of the *PROM1* and *TAPT1* promoter in SEM and RS4;11 cells. ChIP-seq tracks for MLL-N, H3K79me3, H3K27ac, H3K4me1, and H3K4me3, and ATAC-seq at *PROM1* and *TAPT1* in SEM cells, and for MLL-N, H3K79me2, H3K27ac, and H3K4me1, and ATAC-seq at *PROM1* and *TAPT1* in RS4;11 cells. Putative enhancer regions are highlighted in blue. **b** H3K79me3, H3K27ac, H3K4me1 and H3K4me3 ChIP-seq, and ATAC-seq from bone marrow of a child with MLL-AF4 ALL. CD133 expression profile of these cells is shown in Supplementary Fig. 1e (upper panels). Putative enhancer regions are highlighted in blue. **c** Model for the structure of the *PROM1*/*TAPT1* locus, based on Capture-C data. **d** ATAC-seq and H3K27ac ChIP-seq at *PROM1* and *TAPT1* in control (DMSO; orange/green) and EPZ-5676-treated (DOT1Li; gray) SEM cells. Statistically significant decreases in ATAC-seq peaks following DOT1Li are indicated by blue asterisks (FDR < 0.05 from three biological replicates). Histograms show overlay of ATAC-seq and H3K27ac ChIP-seq read counts across the highlighted regions. **e** ATAC-seq in control (DMSO; orange) and EPZ-5676-treated (DOT1Li; gray) RS4;11 cells. Histograms represent overlay of ATAC-seq read counts across the highlighted regions.

To further understand the regulation of *PROM1* by MLL-AF4 we explored the chromatin landscape at *PROM1* by ChIP-seq and ATAC-seq in SEM and RS4;11 cells and primary blast cells from an MLL-AF4 ALL patient, which we confirmed were CD133-positive (Supplementary Fig. 1e, upper panels). Strikingly, in both cell lines (Fig. 3a) and the primary blasts (Fig. 3b) we observed regions marked with H3K27ac, H3K4me1, and H3K79me2/3 and peaks of accessibility within the gene body of *PROM1* and *TAPT1*, indicative of intragenic enhancers (highlighted regions). We have recently demonstrated that KEEs can be functionally important [18] and so we hypothesized that these KEEs may be a common mechanism to upregulate *PROM1* and *TAPT1* in MLL-AF4 ALL.

One characteristic of an active enhancer is that it physically contacts the promoter of the gene it regulates [42–45]. To test whether this was true for these putative enhancers, we performed next generation Capture-C to identify regions interacting with the promoters of *PROM1* and *TAPT1*. In both SEM and RS4;11 cells, the *PROM1* and *TAPT1* KEEs were found in close proximity with their gene promoter, demonstrated by a peak in interaction frequency over the enhancers (Fig. 3a). Interestingly, these interactions were reciprocal, with the *PROM1* intragenic enhancer also contacting the *TAPT1* promoter, and vice versa (Fig. 3a). This strongly argues that these genes exist within the same regulatory domain in SEM and RS4;11 cells, with a potential clover leaf structure whereby both promoters and enhancers may be in close proximity at the same time (Fig. 3c), consistent with their correlated expression in leukemia.

As H3K27ac is commonly used as a marker of active enhancers, a reduction in H3K27ac may be indicative of reduced enhancer activity. We have previously demonstrated that DOT1Li in SEM cells results in a loss of H3K27ac at KEEs [18]. Indeed, there was a striking reduction in H3K27ac at the KEEs of *PROM1* and *TAPT1* following loss of H3K79me2/3 (Fig. 3d). We observed a similar effect by H3K27ac ChIP-qPCR in both SEM and RS4;11 cells (Supplementary Fig. 3e, f). Notably, MLL-AF4 siRNA knockdown produced a similar reduction in H3K27ac (Supplementary Fig. 3g), further suggesting that upregulation of *TAPT1* and *PROM1* by MLL-AF4 is achieved via KEE enhancer function. DOT1Li also led to a reduction in chromatin accessibility at the KEEs of both *PROM1* and *TAPT1* in SEM and RS4;11 cells [18] (Fig. 3d, e) indicative of a reduction in transcription factor binding.

**Fig. 4:**
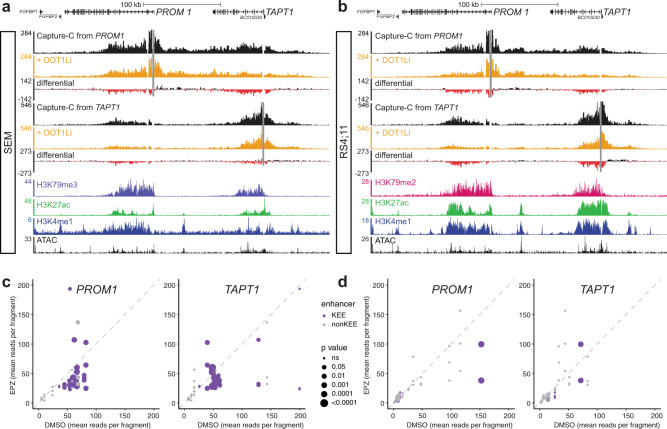
H3K79me2/3 regulates enhancer–promoter interactions at *PROM1* and *TAPT1*. **a, b** Capture-C from the *PROM1* and *TAPT1* promoter (gray bars) in control (DMSO; black) and EPZ-5676-treated (DOT1Li; orange) SEM and RS4;11 cells. Differential tracks show the change in Capture-C signal (black: increases; red: decreases). Representative of three biological replicates. ChIP-seq tracks for H3K79me2/3, H3K27ac, and H3K4me1, and ATAC-seq in SEM and RS4;11 cells. **c**, **d** Statistical analysis of Capture-C-measured changes in interactions between *PROM1* and *TAPT1* promoters and enhancers in SEM (**c**) and RS4;11 (**d**) cells, from three biological replicates. Each circle represents a KEE (H3K79me2/3-marked enhancer element; purple) or non-KEE (enhancer not marked with H3K79me2/3, gray). Size of circle is inversely proportional to the significance of the change in interaction.

We have previously shown that interactions between KEEs and gene promoters are disrupted by DOT1Li, consistent with a loss of enhancer function [18]. Given that the *PROM1* and *TAPT1* KEEs contact both gene promoters (Fig. 3a), we asked whether these interactions were dependent on H3K79me2/3. To test this, we performed Capture-C following DOT1Li in SEM and RS4;11 cells. Strikingly, we observed clear reductions in the interactions between the *PROM1* promoter and the KEEs within both *PROM1* and *TAPT1* in SEM (Fig. 4a) and RS4;11 cells (Fig. 4b). A statistical analysis of enhancer–promoter contact frequency confirmed significant decreases in interaction between the KEEs in *PROM1* and *TAPT1* and both promoters (Fig. 4c, d), arguing that H3K79me2/3 is necessary for enhancer function. Given the decreased expression of *PROM1* following DOT1Li or MLL-AF4 knockdown, this suggests that the loss of enhancer–promoter interactions in the absence of H3K79me2/3 may in part be responsible for the reduction in *PROM1* transcription and subsequent loss of cell surface CD133.

### *PROM1* is polycomb-repressed in CD133− acute leukemia

**Fig. 5:**
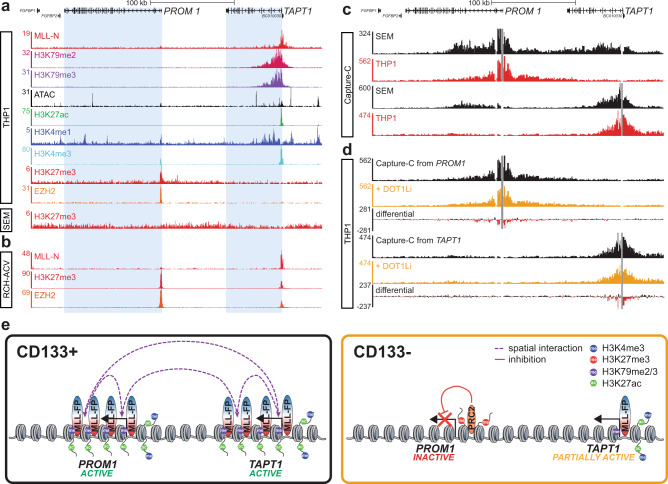
*PROM1* is polycomb-repressed in CD133− leukemia cells. **a** MLL-N, H3K79me2, H3K79me3, H3K27ac, H3K4me1, H3K4me3, H3K27me3 and EZH2 ChIP-seq, and ATAC-seq at *PROM1* and *TAPT1* in THP1 cells. H3K27me3 ChIP-seq at *PROM1* and *TAPT1* in SEM cells. **b** MLL-N, H3K27me3, and EZH2 ChIP-seq at *PROM1* and *TAPT1* in RCH-ACV cells. **c** Comparison of Capture-C tracks from the promoter of *PROM1* and *TAPT1* in SEM (black) and THP1 (red) cells. Gray bars show the Capture-C viewpoint. Tracks are the mean of three biological replicates. **d** Capture-C from the *PROM1* and *TAPT1* promoter in control (DMSO; black) and EPZ-5676-treated (DOT1Li; orange) THP1 cells. Differential tracks show the change in Capture-C signal (black: increases; red: decreases). Tracks represent the mean of three biological replicates. **e** Model for coregulation of *PROM1* and *TAPT1* expression. Left: in CD133-positive MLLr cells, the MLL fusion protein (MLL-FP) binds at the promoters of *PROM1* and *TAPT1* and spreads into the gene body. Recruitment of DOT1L results in elevated H3K79me2/3 levels, facilitating enhancer activity. These KEEs come into proximity with the promoters of both genes, upregulating expression of *PROM1* and *TAPT1*. Right: in CD133-negative MLLr cells, PRC2 binding at the promoter of *PROM1* generates a localized peak of H3K27me3 and disrupts MLL-FP binding at the promoter and gene body, repressing *PROM1* expression. MLL-FP is still able to bind at the promoter of *TAPT1*, but for unknown reasons does not spread into the gene body. The lack of MLL-FP spreading within *PROM1* and *TAPT1* prevents the formation of KEEs, so the expression of neither gene is upregulated.

As observed, not all MLLr leukemias are CD133+ (Fig. 1a, b), and the mechanism of differential *PROM1* regulation in leukemia is unknown. To investigate this, we analyzed THP1 cells, an MLL-AF9 AML cell line that does not express *PROM1*/CD133 (Fig. 1a and Supplementary Fig. 1a). In contrast to SEM cells, we observed no peak of MLL-N (which detects both MLL-AF9 and wild-type MLL) at *PROM1* in THP1 cells or histone modifications associated with enhancers (H3K4me1, H3K27ac, and H3K79me2/3) or promoters (H3K4me3 and H3K27ac) (Fig. 5a). We have previously shown that MLL-AF9 activates target gene expression in THP1 cells by a similar mechanism to MLL-AF4 in SEM cells [17], so we asked why it is unable to bind or activate *PROM1*. Deposition of H3K27me3 by EZH2, the enzymatic component of PRC2, is one mechanism by which genes can be silenced, and is often targeted to developmentally regulated genes [46]. Strikingly, a peak of H3K27me3 and EZH2 was visible at the *PROM1* promoter in place of a strong H3K4me3 peak, whereas no enrichment for H3K27me3 was observed in SEM cells (Fig. 5a, bottom panels). Indeed, we were able to partially relieve the repression of *PROM1* by treatment of THP1 cells with the EZH1/2 and EZH2-specific inhibitors UNC1999 and GSK126, respectively (Supplementary Fig. 4a). These results suggest that *PROM1* repression in THP1 cells is associated with PRC2-mediated H3K27me3, explaining the absence of CD133 on the cell surface.

Interestingly, *TAPT1*, unlike *PROM1*, is expressed in THP1 cells (Supplementary Fig. 1a) and the promoter is bound by MLL-AF9 and marked with active chromatin features (Fig. 5a). In contrast to *PROM1*-expressing SEM and RS4;11 cells (Fig. 3a), however, spreading of the MLL-FP and H3K79me2/3 into the gene body appears to be diminished, associated with reduced H3K27ac (Fig. 5a). This lack of a strong KEE region may in part explain the reduced expression of *TAPT1* in THP1 cells (Supplementary Fig. 1a).

To assess whether PRC2 silencing is a common mechanism for *PROM1* regulation in CD133-negative leukemia and not restricted to MLL-AF9 AML, we analyzed RCH-ACV (non-MLLr Pre-B-ALL) and ML-2 (MLL-AF6 AML) cell lines, which do not express *PROM1* or present CD133 on the cell surface (Fig. 1a and Supplementary Fig. 1a). We observed a peak of H3K27me3 at the promoter of *PROM1* in both RCH-ACV and ML-2 cells (Fig. 5b and Supplementary Fig. 4b), colocalizing with EZH2 in RCH-ACV cells (Fig. 5b and Supplementary Fig. 4c), indicating that this may be a common mechanism to repress *PROM1* in CD133− AML and ALL cells. We also observed no binding of MLL-N at the *PROM1* promoter or gene body in MV4;11, SHI-1, and CCRF-CEM cells (Supplementary Fig. 4d), but detected EZH2 at the promoter in the latter two cell lines (Supplementary Fig. 4c), consistent with a lack of *PROM1* expression in these cells (Supplementary Fig. 1a) due to PRC2 repression. From these data we conclude that the presence of an MLL-FP or EZH2/H3K27me3 at the *PROM1* promoter may determine whether it is expressed in leukemia cells. The reason why *PROM1* is PRC2 repressed in some cell types and expressed in others, and whether the same mechanism drives repression in CD133− primary ALL patient samples is unknown, but may reflect the developmental stage of the cell of origin in different leukemias. However, *PROM1* repression does not reflect the relative expression of *MLL* (*KMT2A*) and *EZH2*, as we observed no correlation between either transcript and *PROM1* in ALL blasts (Supplementary Fig. 4e).

### *PROM1*/*TAPT1* H3K79me2/3 enhancers are inactive in CD133− leukemia

As *PROM1* is repressed in THP1 cells but *TAPT1* is still active we wanted to understand how the topological structure of the locus compared with SEM and RS4;11 cells, where both genes are active and promoter–enhancer interactions are prevalent (Fig. 3a, c). The absence of active KEE marks such as H3K79me2/3, H3K4me1, and H3K27ac, and accessible chromatin at *PROM1* in THP1 cells (Fig. 5a) is consistent with the absence of enhancer activity, so this region would not be expected to interact with the *PROM1* and *TAPT1* promoters. Indeed, using Capture-C we observed no enrichment for interactions between the *PROM1* promoter and the intragenic *PROM1* or *TAPT1* KEEs, in contrast to the high frequency of contacts in SEM cells (Fig. 5c). The low frequency interactions observed spreading from the *PROM1* promoter in THP1 cells are likely due to nonspecific contacts driven by sequence proximity, something that is often observed in Capture-C experiments at inactive loci [21]. A similar effect was observed for interactions with the *TAPT1* promoter, despite the fact that this gene is active (Fig. 5c). The lack of enhancer interactions is consistent with the reduced expression of *TAPT1* in THP1 relative to SEM and RS4;11 cells (Supplementary Fig. 1a). Whilst DOT1Li treatment resulted in some decreases in promoter contacts with local DNA sequences (Fig. 5d), there were no significant effects on interactions with enhancer regions (Supplementary Fig. 4f), further demonstrating the lack of KEE behavior at this locus in THP1 cells. Taken together, these data suggest that PRC2 inactivation of *PROM1* renders the enhancers found within *PROM1* inactive, potentially due to the inability of MLL-FP to bind, which consequently prevents H3K79me2/3 deposition and transcription factor binding. Inactivation of *PROM1* also disrupts any interactions between *PROM1* and the *TAPT1* KEEs (and vice versa), suggesting that inactivation of *PROM1* via PRC2 repression impacts the chromatin structure and activity of the entire domain.

### MLL and PRC2 regulate *PROM1*/CD133 expression during lymphopoiesis

Finally, we sought to test whether the antagonistic relationship between MLL and PRC2 is associated with *PROM1*/CD133 regulation in normal B cell development. We measured CD133 levels on normal fetal BM cells by flow cytometry (Supplementary Fig. 5a). As noted in normal cord blood and postnatal BM [6], CD133 was expressed by the majority of early fetal HSPCs but was significantly reduced on committed B progenitors (CD34+CD19+CD10− PreProB and CD34+CD19+CD10+ ProB progenitors) and almost undetectable on CD34−CD19+ B cells (Supplementary Fig. 5a). Consistent with this, published single-cell RT-qPCR [36] and RNA-seq [34–36] data of normal fetal and adult BM cells demonstrated a restriction of *PROM1* expression to immature CD34+ stem/progenitor cells (Supplementary Fig. 5b, c). Indeed, human cell Atlas single-cell RNA-seq of adult BM cells [38] confirmed restriction of *PROM1* expression to CD34+ cells (Supplementary Fig. 5d). This loss of expression is reflected by the chromatin structure at the *PROM1* locus, where ATAC-seq [34, 36] revealed peaks of accessibility comparable to CD133+ SEM cells in stem and progenitor, but not mature, cells from both fetal and adult BM (Supplementary Fig. 5e).

To ask whether wild-type MLL had a role in *PROM1* expression in HSPCs, we purified CD34+ (CD133+; Supplementary Fig. 5a) and CD34− (CD133−) fetal BM cells and analyzed MLL binding by ChIP-qPCR (Supplementary Fig. 5f). Indeed, MLL binding was only detected at the *PROM1* promoter in the CD34+ cells and not CD34− (CD133−) cells, whilst *TAPT1* and *CDK6* were bound in both progenitor and mature populations (Supplementary Fig. 5f). We confirmed these binding patterns in CD34+ and CD34− fetal liver (FL) cells (Supplementary Fig. 5f). Finally, as in the CD133-negative leukemia cells (Fig. 5a, b), the *PROM1* promoter was marked by H3K27me3 in CD34− FBM and FL, consistent with its repression by PRC2 (Supplementary Fig. 5g). However, in adult BM cells, as for leukemic blasts (Supplementary Fig. 4d), there was no obvious correlation between the expression of *MLL* (*KMT2A*), *EZH2*, and *PROM1* (Supplementary Fig. 5d), suggesting that *PROM1* status is not simply a product of the relative levels of the two proteins.

## Discussion

We demonstrate here that MLL-AF4 directly regulates *PROM1* leading to high expression of CD133 in a major subset of MLLr ALL patients, especially MLL-AF4 and MLL-ENL. MLL-AF4 drives transcriptional upregulation of *PROM1* via H3K79me2/3, which in turn promotes enhancer function. Thus, this mechanism is most likely via aberrant recruitment of DOT1L, resulting in increased H3K79me2/3 levels and increased enhancer–promoter interactions (Fig. 5e), although it is probable that elevated H3K79me2/3 also acts in part by promoting transcription elongation. Interestingly, MLL-AF4 appears to co-regulate high level expression of the neighboring gene *TAPT1* along with *PROM1*. When both genes are expressed, the *PROM1* and *TAPT1* KEEs interact with each other and with both promoters in an H3K79me2/3-dependent manner. Although *TAPT1* is expressed in the absence of *PROM1* transcription, its promoter does not interact with the *PROM1* KEEs, likely driving the lower levels of expression of the *TAPT1* gene (Fig. 5e). Consistent with this, we observe a correlation between expression of *PROM1* and high levels of expression of *TAPT1* in ALL patient samples (Supplementary Fig. 1c). This correlation is not observed in normal hematopoietic cells (Supplementary Fig. 5c, d), consistent with the idea that it is dependent on the MLL-FP-generated KEEs.

One question which arises from our work is why CD133 is expressed in some MLLr leukemias and not others. It is possible that this simply reflects the initial CD133 status of the cell of origin, as many stem and early progenitor cells are known to express CD133 while later progenitor and more mature B cells do not [36, 40, 47, 48]. Another possibility is that the MLL-FP itself has the ability to bind and cause aberrant upregulation of *PROM1*. Indeed, the observation that not all MLL-FPs are associated with high levels of CD133 suggests that the activity of the fusion partner may be important for its ability to bind at *PROM1*. We note that we do not observe binding of wild-type MLL at *PROM1* in CD133− cells (Fig. 5a, b and Supplementary Figs. 4d, 5f). Alternatively, differences in the chromatin state at *PROM1* may render the environment permissive for MLL-FP spreading only in certain target cell types, so leukemias that initiate in a *PROM1*-repressed environment may not be capable of reactivating the locus. For instance, in CD133− leukemia cell lines such as THP1 and RCH-ACV, the promoter of *PROM1* is bound by EZH2 (the enzymatic component of PRC2) and marked with H3K27me3, suggesting that the gene is polycomb-repressed.

What determines whether MLL-FP or PRC2 becomes stably bound at *PROM1*? Polycomb and Trithorax group proteins (such as MLL) are known to be antagonistic [49], so stable PRC2 binding at the promoter may prevent MLL-FP association, and vice versa (both can recognize unmethylated CpG islands [49]). In this model, in CD133− leukemias PRC2 wins this competition, rendering *PROM1* repressed. In contrast, in MLLr CD133+ leukemias MLL-FP binding may be preferentially stabilized over PRC2 and therefore *PROM1* is activated. In past work, we have noted that the spreading of MLL-AF4 binding into the gene body, as observed at *PROM1*, is associated with particularly high levels of MLL-AF4-mediated transcription [17], but it was not known whether spreading initiated at the promoter. The fact that when *PROM1* is repressed the MLL-FP is also absent from the body of the gene (even though PRC2 is not bound here) suggests that spreading may nucleate from the promoter. It should be noted that non-MLLr leukemias can also be CD133+ (see Fig. 1b and [40, 50, 51]), as are many stem cells [36, 40], in the absence of an MLL-FP to drive expression. We identified MLL binding at the *PROM1* promoter in normal fetal CD34+ stem and progenitor cells (Supplementary Fig. 5f), arguing that wild-type MLL plays a role in *PROM1* expression, and there are likely other oncogenic mechanisms for *PROM1* upregulation.

Our work here suggests that early stem/progenitor cell expression of *PROM1*/CD133 eventually becomes repressed by PRC2 in more mature cells. This raises an important question—do MLL-FPs such as MLL-AF4 overcome this repressive environment and aberrantly activate *PROM1*/CD133 or does the transformation event occur in a progenitor cell that still displays *PROM1*/CD133 expression? Our data cannot fully answer this question, but an emerging model in the field suggests that PRCs are responsive to, rather than instructive for, gene repression [49, 52, 53]. What this might indicate is that *PROM1*/CD133 is normally repressed by various developmental signals (e.g., binding of repressive transcription factors) that eventually lead to PRC-mediated repression. If MLL-FPs are able to bind to *PROM1* in a cell type that has not completely repressed the locus, this could lead to a disruption of the normal repressive machinery and reactivation of the gene. The fact that inhibiting PRC2 activity partially reactivates the locus (Supplementary Fig. 4a) suggests that, even in CD133− cells, there is some low-level ability to activate the locus that is normally suppressed.

The mechanism of *PROM1* regulation is of relevance when considering the fetal cell of origin of infant MLL-AF4 ALL, which invariably originates in utero. CD133 is expressed during normal fetal hematopoiesis primarily in hematopoietic stem cells (HSCs) and early progenitors (multipotent progenitors (MPPs) and lymphoid-primed MPPs) [36, 40]. This is consistent with a permissive environment for MLL-AF4 binding at *PROM1*, if the leukemia was initiated in any of these cell types. However, infant MLL-AF4 ALL shares some characteristics with a more developed B cell progenitor [20] such as fetal BM PreProB progenitors [36], suggesting this could be the cell of origin [33, 54]. In these cells *PROM1* is much more weakly expressed and may be in the process of PRC2 repression, but MLL-AF4 induction could antagonize this process by competing for binding at the promoter. Notably, the chromatin environment at *PROM1* remains accessible in PreProB progenitor cells, despite the low expression level, and therefore may be permissive for MLL-AF4 binding. Thus, the balance between Trithorax and polycomb group proteins at *PROM1* upon leukemia initiation may render CD133 either highly expressed or absent.

One important question is whether CD133 could be a useful therapeutic target. Since CD133 is expressed on both fetal and adult HSCs, it may be difficult to target CD133+ blasts without impacting normal HSCs [40]. One approach that has been proposed is to combine CD133 targeting with a second marker found on ALL blasts such as CD19 [4]. An “AND gate” strategy that requires dual target engagement for activity would spare normal HSCs. However, such a strategy is contingent on the inability of the leukemia cells to downregulate expression of either marker. Of note, we show here that continuous expression of CD133 is essential for the growth of SEM and RS4;11 cells, as it functions at least in part by promoting cell cycle progression and suppressing apoptosis. This suggests that CD133+ leukemia cells may be addicted to CD133 expression (whilst CD133− blasts tolerate its absence). However, some long-term repopulating ALL cells have been shown to be CD133+/CD19−, complicating the use of an “AND gate” strategy in this way [51]. In the large subset of MLLr ALL patients where *PROM1*/CD133 is co-expressed with CD19, CD133/CD19 dual AND gate targeting could be a promising targeted therapeutic approach in difficult-to-treat infants, children, and adults. To be truly effective in a wide range of patients, combination therapy will be required, with other approaches such as BCL2 inhibition [55, 56] or targeting other MLLr-specific cell surface receptors, such as NG2 [57].

## Supplementary information

Supplementary Figures and Tables

## Data Availability

All high-throughput data have been deposited in the Gene Expression Omnibus (GEO) under accession number GSE135026. Accession numbers for datasets used from previous publications can be found in Supplementary Table 3.

## Code availability

The ChIP-seq and ATAC-seq sequence mapping pipeline can be accessed at https://github.com/Hughes-Genome-Group/NGseqBasic/releases. Capture-C analysis code is available from https://github.com/Hughes-Genome-Group/CCseqBasicF/releases.
