# Supplementary material for: H3K79me2/3 controls enhancer–promoter interactions and activation of the pan-cancer stem cell marker PROM1/CD133 in MLL-AF4 leukemia cells
Source: Leukemia. 2020 Apr 2;35(1):90–106. doi: 10.1038/s41375-020-0808-y (PMC7787973; doi:10.1038/s41375-020-0808-y)
Supplement: Supplementary file 1 — Supplementary Figures and Tables [file 41375_2020_808_MOESM1_ESM.pdf]

H3K79me2/3 controls enhancer promoter interactions and activation of the pan-cancer stem cell marker *PROM1*/CD133 in MLL-AF4 leukemia cells

Godfrey et al.

Description: Supplementary Figures and Legends, Supplementary Tables

Supplementary Figure 1

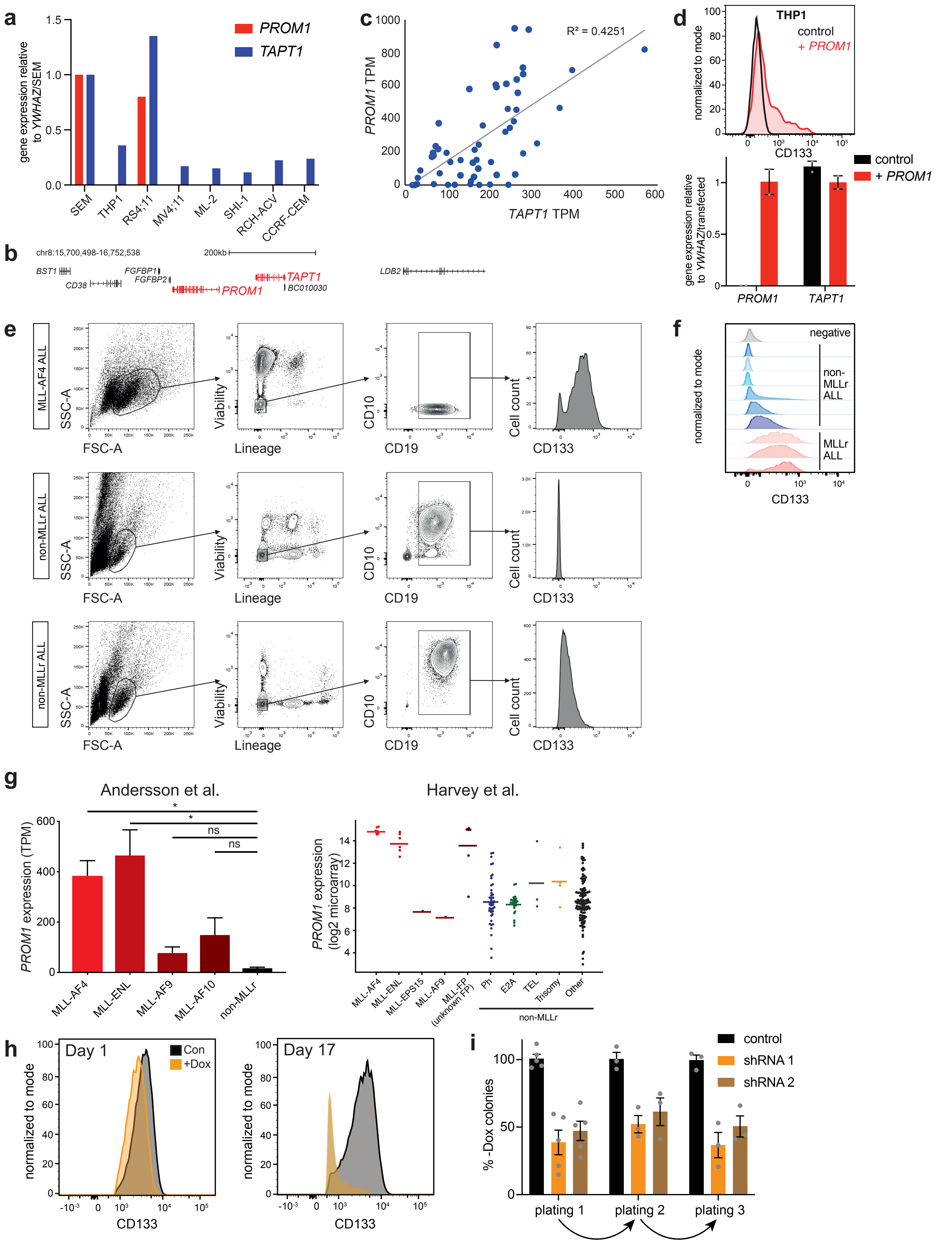

Supplementary Figure 1. CD133 expression is essential for MLL-AF4 ALL cell growth

(a) qRT-PCR of *PROM1* (red) and *TAPT1* (blue), relative to *YWHAZ*, in the indicated leukemia cell lines, normalized to levels in SEM cells. (b) Gene map showing the relative genomic locations of *PROM1* and *TAPT1*. (c) Correlational analysis between *PROM1* and *TAPT1* expression in the Andersson *et al* dataset (32) $R^2 = 0.4251$. The trend line is shown in gray. (d) Transfection of THP1 cells with a *PROM1* overexpression plasmid. *Above*: Flow cytometry analysis of CD133 levels on control (black) and *PROM1*-transfected (red) THP1 cells after 72h. Representative of two biological replicates. *Below*: qRT-PCR analysis of *PROM1* and *TAPT1* expression in control (black) and *PROM1*-transfected (red) THP1 cells after 72h, relative to *YWHAZ*, normalized to levels in transfected cells. Error bars show s.d. of two biological replicates. (e) Representative plots showing simplified gating strategy for CD133 flow cytometry analysis of bone marrow samples from MLL-AF4+ ALL patient (top row) and non-MLLr ALL patients (middle and bottom row). The MLL-AF4+ sample shown was subsequently analyzed by ChIP-seq and ATAC-seq (Fig. 3b). Lineage: CD2, CD3, CD14, CD16, CD56, CD235a. (f) Flow cytometry analysis of CD133 levels in three MLLr ALL patients (red) and six non-MLLr ALL patients (blue) compared to unstained negative control (gray). (g) Expression of *PROM1* from two independent published datasets, grouped by MLL-FP. *Left*: RNA-seq expression of *PROM1* in MLL-FP ALL and non-MLLr ALL patient blasts (32), * = $p<0.05$; *right*: Microarray expression of *PROM1* in MLL-FP and non-MLLr ALL patient blasts (31). (h) Flow cytometry analysis showing CD133 level in *PROM1* shRNA SEM cell line 2 in control (black) and induced (orange) conditions at day 1 (24h doxycycline treatment) and day 17 (following colony assay). Histograms are representative of three replicates. (i) Serial replating of control and *PROM1* shRNA SEM cells. Data are presented as the percentage of colonies growing in the presence of 0.5 µg/ml doxycycline relative to the same cell line in the absence of doxycycline; n=5 (plating 1), n=3 (platings 2 and 3); error bars show s.e.m.

Supplementary Figure 2

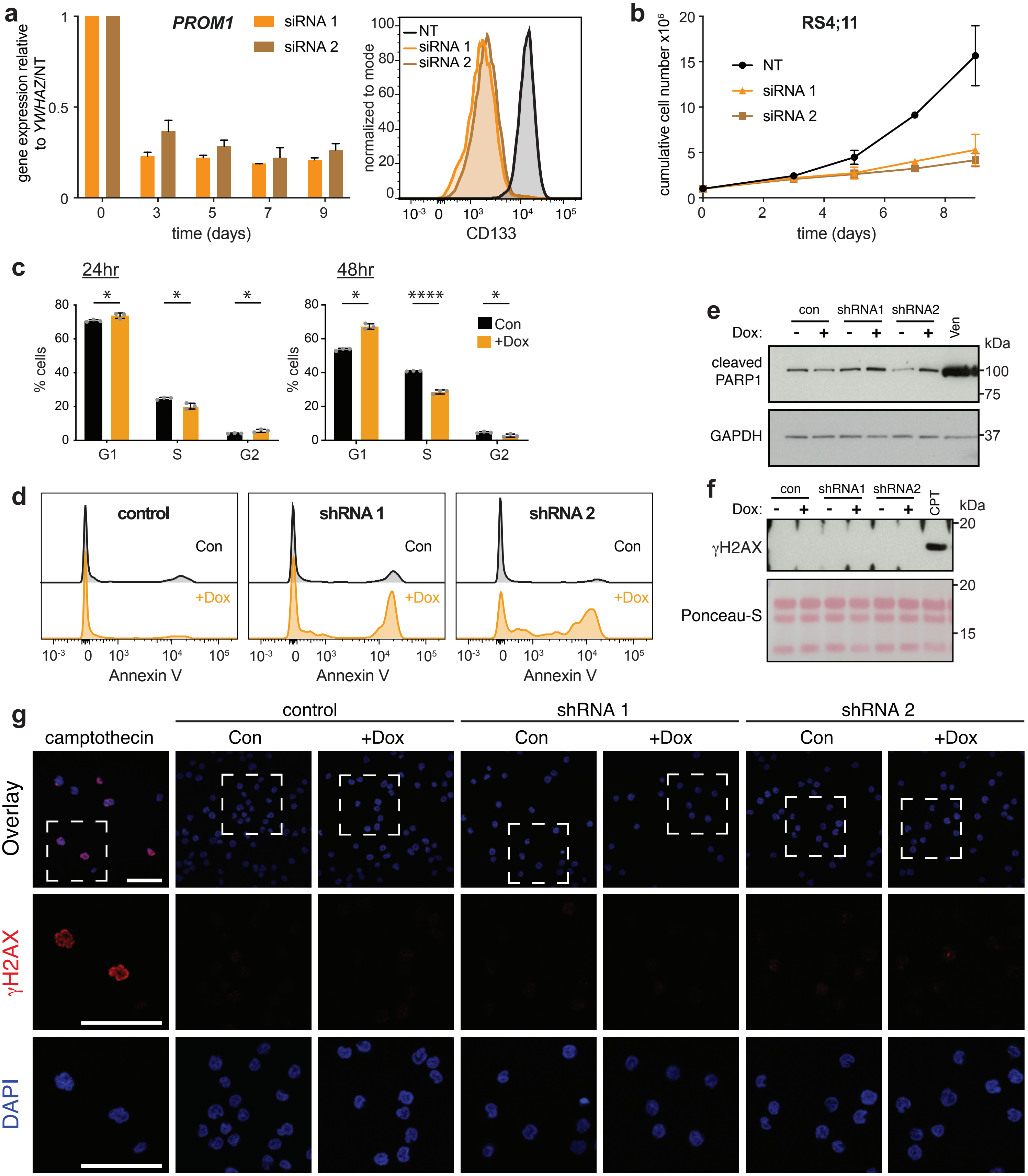

Supplementary Figure 2. *PROM1* knockdown disrupts cell cycle and induces apoptosis (a) *PROM1* siRNA transfection of RS4;11 cells results in a reduction of CD133/*PROM1*. *Left*: RS4;11 cells were transfected with one of two *PROM1*-targeting siRNAs or non-targeting (NT) siRNA, retransfected after 72h and subsequently every 48h. *PROM1* expression was measured by qRT-PCR, normalized to *YWHAZ* and relative to NT-transfected cells. Error bars show s.d., n=2. *Right*: Flow cytometry analysis of CD133 levels on RS4;11 cells 72h after siRNA transfection. (b) Cumulative growth of RS4;11 cells transfected with siRNA as in (a). Error bars show s.d., n=2. (c) Cell cycle analysis using flow cytometry in 24 h and 48 h control (black) and induced (orange) *PROM1* shRNA 1 SEM cells. Error bars represent s.e.m. from three biological replicates, **** = $p<0.0001$, * = $p<0.05$. (d) Flow cytometry analysis of Annexin V staining of viable control or *PROM1* shRNA SEM cell lines, either untreated (Con) or treated with 0.5 µg/ml doxycycline for 72h (+Dox), n=1. (e) Western blot of cleaved PARP and GAPDH levels in control or *PROM1* shRNA SEM cell lines, either untreated (-) or treated with 0.5 µg/ml doxycycline for 72h (+), representative of two replicates. Ven: 0.5 µM venetoclax treatment for 24h. (f) Western blot of γH2AX in control or *PROM1* shRNA SEM cell lines, either untreated (-) or treated with 0.5 µg/ml doxycycline for 72h (+). Representative of two replicates. CPT: 1 µM camptothecin treatment for 1h. (g) Immunofluorescence analysis of γH2AX in control or *PROM1* shRNA SEM cell lines, either untreated (Con) or treated with 0.5 µg/ml doxycycline for 72h (+Dox), or 1 µM camptothecin for 1 h. Dashed box indicates region shown expanded in lower two panels. Scale bar length 50 µm. Representative view of one replicate.

Supplementary Figure 3

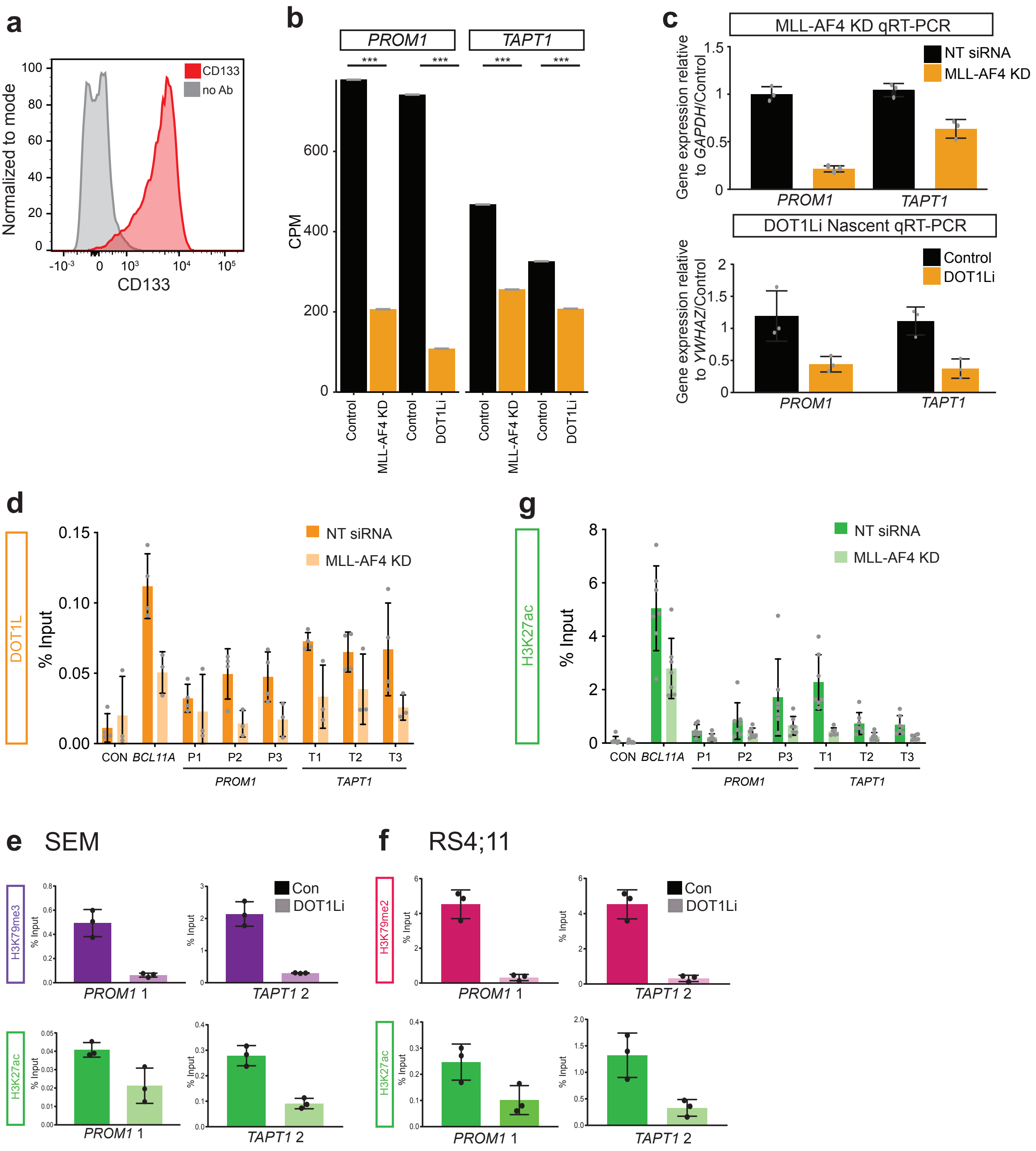

Supplementary Figure 3. ***PROM1* is regulated by MLL-AF4 and H3K79me2/3-marked enhancers** (a) Flow cytometry analysis showing CD133 expression in the primograft sample used for ChIP-seq in Fig. 2a-b. CD19-positive cells were analyzed, with a sample without anti-CD133 antibody (no Ab) used as a control, n=1. (b) *PROM1* and *TAPT1* transcript levels from MLL-AF4 knockdown and DOT1Li nascent RNA sequencing in control (black) and MLL-AF4 knockdown/EPZ-5676 (orange). Values given are counts per million (CPM); error bars represent s.e.m. of three biological replicates, *** = $p<0.001$. (c) *Upper*: qRT-PCR for *PROM1* and *TAPT1* in control (black) and MLL-AF4 siRNA knockdown (orange) SEM cells, relative to *GAPDH* and normalized to control samples. *Lower*: Nascent RNA qRT-PCR for *PROM1* and *TAPT1* in control (black) and DOT1Li treated (orange) SEM cells, relative to *YWHAZ* and normalized to control samples. Error bars represent s.e.m. from three biological replicates. (d) DOT1L ChIP-qPCR in control (orange) and MLL-AF4 siRNA knockdown (light orange) SEM cells at a negative control locus (CON), *BCL11A* and several regions of *PROM1* and *TAPT1* in SEM cells (primer locations indicated in Fig. 3a). Error bars represent s.e.m. from five biological replicates. (e) H3K79me3 and H3K27ac ChIP-qPCR at *PROM1* and *TAPT1* in control (dark shade) or DOT1Li (light shade) SEM cells. Error bars represent s.e.m. from five biological replicates. (f) H3K79me2 and H3K27ac ChIP-qPCR at *PROM1* and *TAPT1* in control (dark shade) or DOT1Li (light shade) RS4;11 cells. Error bars represent s.e.m. from three biological replicates. (g) H3K27ac ChIP-qPCR in control (green) and MLL-AF4 siRNA knockdown (light green) SEM cells at a negative control locus (CON), *BCL11A* and several regions of *PROM1* and *TAPT1* in SEM cells (primer locations indicated in Fig. 3a). Error bars represent s.e.m. from five biological replicates.

Supplementary Figure 4

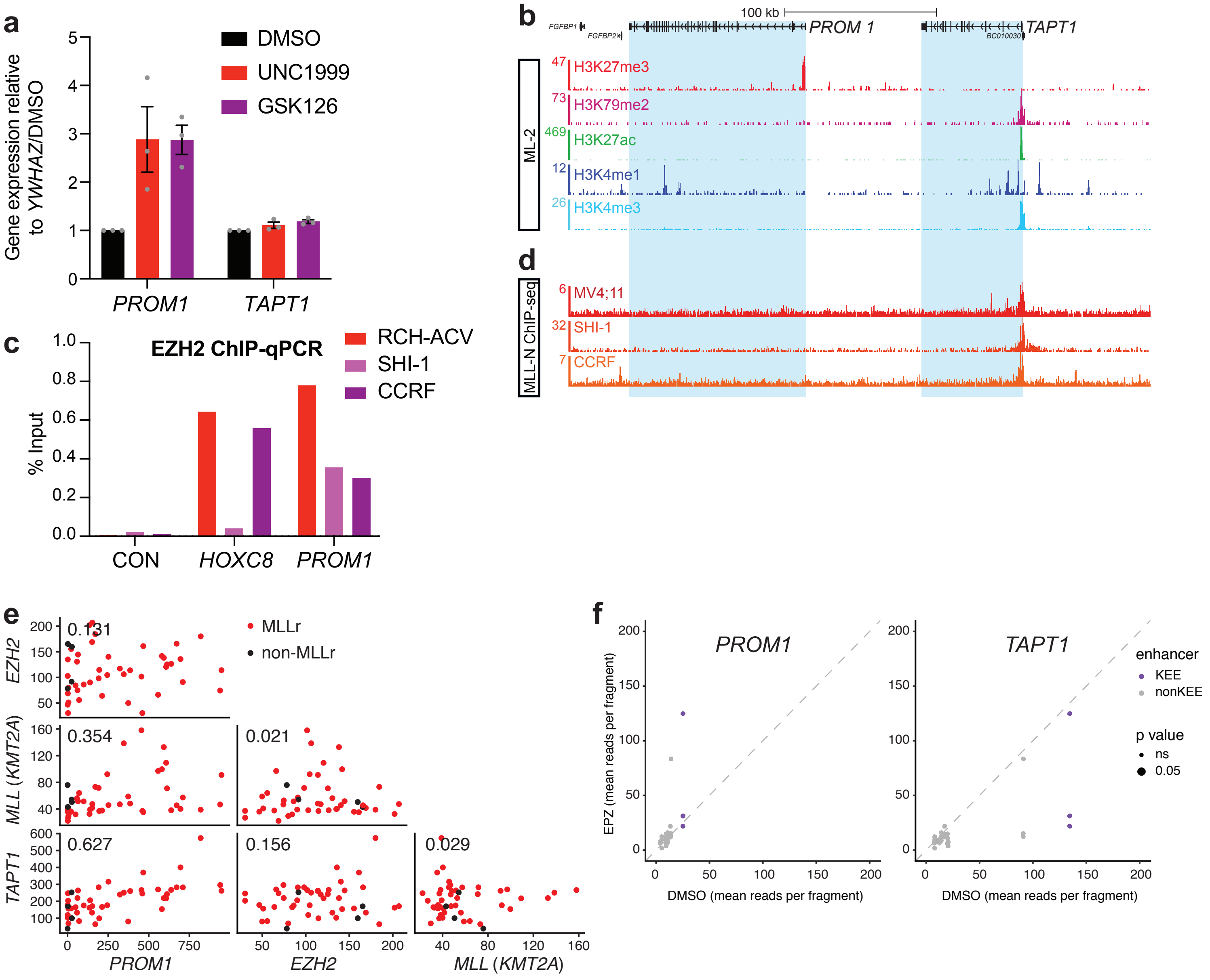

Supplementary Figure 4. *PROM1* is polycomb-repressed in CD133- leukemia cells (a) qRT-PCR analysis of *PROM1* and *TAPT1* expression in THP1 cells treated with 5 μM UNC1999 or 2 μM GSK126 for 5 days, normalized to *YWHAZ* and relative to DMSO-treated cells. Error bars represent s.e.m. of three biological replicates. (b) H3K27me3, H3K79me2, H3K27ac, H3K4me1 and H3K4me3 ChIP-seq at *PROM1* and *TAPT1* in ML-2 cells, n=1. (c) ChIP-qPCR for EZH2 in RCH-ACV, SHI-1 and CCRF-CEM cells, n=1. (d) MLL-N ChIP-seq at *PROM1* and *TAPT1* in MV4;11, SHI-1 and CCRF-CEM cells, n=1. (e) Correlational analysis between *PROM1*, *TAPT1, MLL* and *EZH2* expression in B-ALL blasts (28). Numbers indicate the Pearson correlation coefficient for each pairwise comparison. (f) Statistical analysis of Capture-C-measured changes in interactions between *PROM1* and *TAPT1* promoters and enhancers in THP1 cells, from three biological replicates. Each circle represents a KEE (H3K79me2/3-marked enhancer element; purple) or nonKEE (enhancer not marked with H3K79me2/3; gray). Size of circle is inversely proportional to the significance of the change in interaction.

Supplementary Figure 5

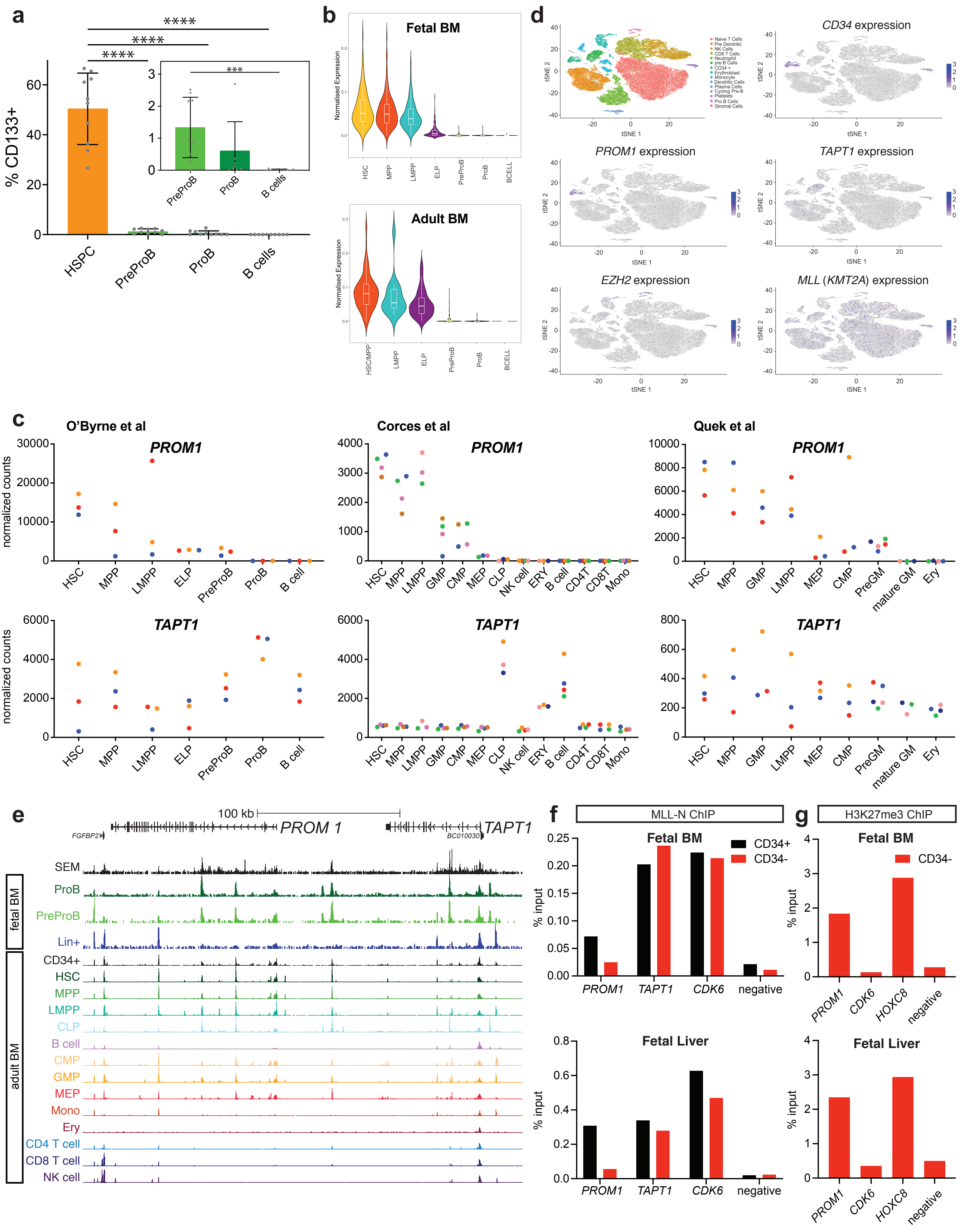

Supplementary Figure 5. *PROM1* expression during hematopoiesis correlates with MLL binding (a) Flow cytometry analysis showing the percentage of CD133+ cells in human 2nd trimester fetal BM HSPCs (Lin-CD34+CD38-), PreProB progenitors (Lin2-CD34+CD19+CD10-), ProB progenitors (Lin2-CD34+CD19+CD10+), and mature B cells (CD34-CD19+). Graphs represent mean, error bars show s.e.m. from ten biological replicates. Inset shows magnified scale for PreProB, ProB and B cells. Lin cocktail: CD2, CD3, CD14, CD16, CD56, CD19, CD235a; Lin2 cocktail: CD2, CD3, CD14, CD16, CD56, CD235a. (b) Single cell RT-qPCR data showing *PROM1* expression in fetal and adult BM populations, taken from (36). (c) RNA-seq analysis of *PROM1* and *TAPT1* expression in adult BM populations, taken from (34-36). Colors represent different donors. (d) Single-cell RNA-seq data from the Human Cell Atlas (38) was used to generate a tSNE plot. Relative expression of the indicated genes is plotted as a heatmap. (e) ATAC-seq of sorted populations from fetal BM (36) and adult BM (34) at the *PROM1*/*TAPT1* locus. (f) ChIP-qPCR for MLL in CD34+ and CD34- fetal BM (*above*) and fetal liver (*below*) cells, n=1. (g) ChIP-qPCR for H3K27me3 in CD34- fetal BM (*above*) and fetal liver (*below*) cells, n=1.

Supplementary Table 1 Antibodies used in this study

Target	Application	Clone	Catalog Number	Company
MLL-N	ChIP-qPCR		A300-086A	Bethyl
AF4-C	ChIP-qPCR		ab31812	Abcam
H3K79me2	ChIP-qPCR		ab3594	Abcam
H3K79me3	ChIP-seq, ChIP-qPCR		C15410068	Diagenode
H3K27ac	ChIP-seq, ChIP-qPCR		C15410196	Diagenode
H3K4me1	ChIP-seq		pAb-194-050	Diagenode
H3K4me3	ChIP-seq		pAb-003-050	Diagenode
H3K27me3	ChIP-seq, ChIP-qPCR		07-449	Millipore
EZH2	ChIP-seq, ChIP-qPCR		5246/4	Cell Signaling Technology
DOT1L	ChIP-qPCR		A300-953A	Bethyl
Cleaved PARP1	Western blotting		5625	Cell Signaling Technology
γH2AX	Western blotting		9718	Cell Signaling Technology
GAPDH	Western blotting		A300-641A	Bethyl
CD2-PerCP-Cy5.5	FACS (Oxford)	RPA-2.10	300215	Biolegend
CD3-PerCP-Cy5.5	FACS (Oxford)	okt3	317336	Biolegend
CD14-PerCP-Cy5.5	FACS (Oxford)	M5E2	301824	Biolegend
CD16-PerCP-Cy5.5	FACS (Oxford)	3G8	302027	Biolegend
CD56-PerCP-Cy5.5	FACS (Oxford)	HCD56	318322	Biolegend
CD235a-PerCP-Cy5.5	FACS (Oxford)	HIR2	306614	Biolegend
CD34-PE-Cy7	FACS (Oxford)	4H11	25-0349-42	ThermoFisher Scientific
CD38-BV605	FACS (Oxford)	HIT2	551400	Biolegend
CD45-AF700	FACS (Oxford)	2D1	56-9459-42	ThermoFisher Scientific
CD133-PE	FACS (Oxford)	AC133	130-098-826	Miltenyi
CD19-APC	FACS (Oxford)	HIB19	302212	Biolegend
CD10-FITC	FACS (Oxford)	ebioCB-CALLA	11-0106-42	ThermoFisher Scientific
CD20-ef450	FACS (Oxford)	2H7	48-0209-41	ThermoFisher Scientific
Annexin V-APC	FACS (Oxford)		88-8007-72	ThermoFisher Scientific
CD133-APC	FACS (Oxford Primograft)	AC133	130-098-826	Miltenyi

CD19-FITC	FACS (Oxford Primograft)	HIB19	11-0199042	ThermoFisher Scientific
CD38-FITC	FACS (GOSH)	HB-7	340909	BD
CD10-PE-Cy7	FACS (GOSH)	HI1Da	341112	BD
CD133-PE	FACS (GOSH)	AC133	130-113-108	Miltenyi
CD34-PerCp	FACS (GOSH)	8G12	345903	BD
CD20-APC-H7	FACS (GOSH)	L27	641414	BD
CD45-V450	FACS (GOSH)	2D1	642275	BD
CD24-BV510	FACS (GOSH)	ML5	563035	BD
CD19-APC	FACS (GOSH)	SJ25C1	345791	BD
CD117-PE-Cy7	FACS (GOSH)	104D2	339217	BD
CD33-APC	FACS (GOSH)	P67.6	345800	BD
CD15-BV510	FACS (GOSH)	W6D3	563141	BD
CD15-Krome Orange	FACS (GOSH)	80H5	B01176	Beckman
CD11b-APC-H7	FACS (GOSH)	ICRF44	560914	BD

Supplementary Table 2 qPCR primers used in this study

Primer name	Forward Sequence/ Taqman probe code	Reverse Sequence
YWHAZ	Hs03044281_g1	
PROM1	Hs01009257_m1	
TAPT1	Hs00538015_m1	
BCL11A	ACACCCAGTGCCCAGAATTG	CGCGGGTCCTGAGATTCATT
Negative control region	GGCTCCTGTAACCAACCACTACC	CCTCTGGGCTGGCTTCATTC
PROM1 1	CCCTTTATGGGTTCGGGAAATA	GAGAACAGTGCCTGGAAGATAG
PROM1 2	GGTGACACAATTCAACCCATAAC	CATGGGTCTTTAGTTGGTGTAGTA
PROM1 3	CCACAACCCTCAATTTCCTTTC	GACACAAGCACTGGGTCATA
PROM1 promoter (H3K27me3/MLL ChIP)	CAGAAAGGTCAGTCGCCTTAG	GGAGGTGGTTTGGAGGTAAAG
TAPT1 1	CTGGAAGCTTGAGGTGAGAAG	GGGCTATCAGGGAAGTGTAATG
TAPT1 2	ACAAGCAGGGTGTGACAAG	GAGAAGCAGGAAAGCCAGTAG
TAPT1 3	TCCTCCCTTCGGTCTGATTTA	TTATTGTCCCGTTTCCTCACC
TAPT1 promoter (H3K27me3/MLL ChIP)	CGGACTTTCCACACTGTCTT	CCCAAGTAAAGCGACAAAGTAAG
HOXC8	AGACTTCTTCCACCACGGCAC	TAAGCGAGCACGGGTTCTGC
CDK6	TCGAAGCGAAGTCCTCAACA	GCTTGGGCAGAGGCTATGTA

Supplementary Table 3 Publicly available datasets used in this study

Data type	Cell type	Sample	GEO/ENA/EGA accession number
ATAC-seq	SEM	DMSO 7d	GSE117865
ATAC-seq	SEM	EPZ-5676 7d	GSE117865
ATAC-seq	RS4;11	DMSO 7d	GSE117865
ATAC-seq	RS4;11	EPZ-5676 7d	GSE117865
ATAC-seq	Fetal bone marrow	PreB, PreProB, Lin+ MNCs	GSE122989
ATAC-seq	Adult bone marrow	CD34+, HSC, MPP, LMPP, CLP, B cell, CMP, GMP, MEP, Monocyte, Erythrocyte, CD4 and CD8 T cells, NK cells	GSE74912
Capture-C	SEM	DMSO 7d	GSE117865
Capture-C	SEM	EPZ-5676 7d	GSE117865
Capture-C	RS4;11	DMSO 7d	GSE117865
Capture-C	RS4;11	EPZ-5676 7d	GSE117865
Capture-C	THP1	DMSO 7d	GSE117865
Capture-C	THP1	EPZ-5676 7d	GSE117865
ChIP-seq	SEM	H3K4me1	GSE74812
ChIP-seq	SEM	H3K4me3	GSE74812
ChIP-seq	SEM	H3K27ac	GSE74812
ChIP-seq	SEM	H3K79me2	GSE74812
ChIP-seq	SEM	H3K79me3	GSE74812
ChIP-seq	SEM	H3K79me3 DMSO 7d (reference-normalized)	GSE117865
ChIP-seq	SEM	H3K79me3 EPZ-5676 7d (reference-normalized)	GSE117865
ChIP-seq	SEM	H3K27ac DMSO 7d	GSE117865
ChIP-seq	SEM	H3K27ac EPZ-5676 7d	GSE117865
ChIP-seq	SEM	MLL-N	GSE74812
ChIP-seq	SEM	AF4-C	GSE74812
ChIP-seq	RS4;11	H3K4me1	GSE71616
ChIP-seq	RS4;11	H3K27ac	GSE71616
ChIP-seq	RS4;11	H3K79me2	GSE38403
ChIP-seq	RS4;11	MLL-N	GSE38403
ChIP-seq	RS4;11	AF4-C	GSE38403
ChIP-seq	THP1	H3K4me1	GSE117865
ChIP-seq	THP1	H3K4me3	GSE117865
ChIP-seq	THP1	H3K27ac	GSE117865
ChIP-seq	THP1	H3K79me2	GSE117865
ChIP-seq	THP1	MLL-N	GSE83671

ChIP-seq	ML-2	H3K4me3	GSE95511
ChIP-seq	ML-2	H3K27ac	GSE95511
ChIP-seq	ML-2	H3K27me3	GSE95511
ChIP-seq	ML-2	H3K79me2	GSE83671
ChIP-seq	SHI1	MLL-N	GSE95511
ChIP-seq	MV4;11	MLL-N	GSE83671
ChIP-seq	CCRF-CEM	MLL-N	GSE83671
ChIP-seq	RCH-ACV	MLL-N	GSE83671
ChIP-seq	CD34+ cordblood	FLAG-MLL-Af4	GSE84116
ChIP-seq	Primograft	MLL-N	GSE83671
ChIP-seq	Primograft	AF4-C	GSE83671
Nascent RNA-seq	SEM	siMM (NT siRNA)	GSE85988
Nascent RNA-seq	SEM	siMA6 (MLL-AF4 KD siRNA)	GSE85988
Nascent RNA-seq	SEM	DMSO 7d	GSE83671
Nascent RNA-seq	SEM	EPZ-5676 7d	GSE83671
RNA-seq	iALL blasts	Agraz-Doblas *et al* dataset	PRJEB23605 (European Nucleotide Archive)
RNA-seq	iALL blasts	Andersson *et al* dataset	EGAS00001000246 (European Genome-phenome Archive)
RNA-seq	Fetal bone marrow	HSC, MPP, LMPP, ELP, PreProB, ProB, B cells	GSE122982
RNA-seq	Adult bone marrow	HSC, MPP, GMP, LMPP, MEP, CMP, PreGM, mature GM, Erythrocyte	ArrayExpress accession no. E-MTAB-2672
RNA-seq	Adult bone marrow	HSC, MPP, LMPP, GMP, CMP, MEP, CLP, B cell, Monocyte, Erythrocyte, CD4 and CD8 T cells, NK cells	GSE74246
single-cell RNA-seq	Adult bone marrow	>100,000 single cells analyzed	http://www.altanalyze.org/ICGS/HCA/Viewer.php
Microarray data	ALL blasts	Harvey *et al* dataset	GSE11877
